# Address to the Graduating Class of Ohio Dental College, March 1, 1871

**Published:** 1871-04

**Authors:** F. H. Rehwinkle


### DENTAL REGISTER.

VOL. XXV.] APRIL, 1871. [No. 4.

### Communications.

### ADDRESS TO THE GRADUATING CLASS OF OHIO DENTAL COLLEGE, MARCH 1, 1871.

BY DR. F. H. REHWINKLE.

*Gentlemen of the Graduating Class:*

On occasions like the present, it is, I believe, customary for the one chosen by your faculty to deliver the valedictory to begin by expressing regret that the choice had not fallen upon one better fitted for the duty—one practiced in moulding thought into flowing and eloquent language. Gladly would I make an exception to this rule, but I am compelled by candor to acknowledge myself guilty of a weakness in accepting this honor, instead of yielding to my first impulse, which was to urge upon the gentlemen of your faculty the expediency of selecting from among the "shining lights" of our profession one who could have better honored this, the twenty-fifth commencement of our college.

Regrets at this late day being useless, be pleased to accept a few thoughts and ideas suggested by this, to you, momentous event. It is not my purpose to weary you with a vain attempt at eloquence. I belong not to the favored few at whose side "little angels stand" whispering words at once thrilling, flashing, and burning with the fire of eloquence. I *may* have wooed them, but failure is my record. Whatever I offer you, therefore, can only be said in a plain common sense way, clothed in homespun garb.

Your being assembled here to-night brings vividly to my mind the scenes of years ago, the time when I with others occupied your position; it brings back, too, the high hopes and aspirations, sanguine expectations and earnest resolves.

Gentlemen, you have arrived at a crisis in your lives; and it will depend as much upon your moral strength of character as upon the possession of the requisite professional qualifications, whether you will rise above mediocrity and to eminence in your chosen avocation, or whether you will merely occupy a space. You have made choice of a profession which will task all the strength within you; a field of labor at once complicated and without limit.

As a specialty of medicine and surgery, operative dentistry involves a necessity for a general knowledge of the whole science of the healing art. I call it a specialty of medicine and surgery. Thirty or forty years ago this would have been deemed presumptuous. Its relations to medical science had not then been so universally studied, and were understood but by comparatively few. To the noble and energetic spirit of these latter, combined with their successful efforts, *this* claim stands to-day a fact, accomplished and undisputed, and our profession is now elevated to its proper position and its rights recognized. To those who have been chiefly instrumental in accomplishing this end, we owe all thanks. In view of these things it follows that the duties that you must assume on entering into actual practice are commensurately great, and your qualifications should be unquestionable. As a dental surgeon you must not only be *thoroughly at home* in the knowledge of the anatomy of the head and adjacent parts, but should have a perfect understanding of anatomy as a whole. You can not be a good pathologist without being well versed in physiology, whilst without chemistry you would be like a mariner without a compass. In short, my young friends, your professional education must be very thorough. Added to this you will require a high degree of mechanical aptitude, combined with manipulative skill, to perform difficult and intricate mechanico-surgical operations upon the living and sensitive tissues of the mouth.

"Are you well qualified" for these duties and responsibilities?

Have you acquired the necessary proficiency in all the various departments of science as connected with your profession, and have you taken the initiatory lessons in the collateral branches appertaining thereto?

Your faculty answers for you in the affirmative, and gives you an indorsement which speaks well for your attainments. We can not ask a better guarantee than that of your being graduates of the Ohio Dental College, and worthy pupils of able masters.

This pleasant event which calls us together, with many of the elder members of our profession, must stir up memories of "Auld Lang Syne," and perhaps regret, aye, envy may mingle with their thoughts to-night. Consider, my young friends, how natural it must be for our old and honored pioneers, some of whom are, per chance, within the sound of my voice, to institute comparisons with the *then* and *now*. Think for a moment what a rough and thorny path they had to travel. There were no colleges of dentistry for them. No associations or facilities for the exchange of thought, ideas, and modes of practice. Isolated, each one, with what now appears to us but crude preparatory instructions, obtained in the office and laboratory of their preceptors, and this almost under the seal of secrecy. No dental literature, excepting a few collections of instructions appertaining mostly to the mechanical part of dentistry. Treading wearily and alone, yet ardently hoping and laboring to bring about a better state of things. That their labors have been crowned with abundant success, witness this night's exercises. Do you, then, think it strange that their retrospection to night should be accompanied by wistful regret that such facilities for acquiring a professional education were never within their reach? Is it a matter of wonder that they should almost envy you your good fortune in being the recipients of all the patiently gathered and carefully assorted facts, and of the concentrated and purified results of an experience of years? Well might they ask, both of you and me, do you fully realize and appreciate the opportunities and privileges which the present age offers to the disciple of art and science?

Indeed, we might all of us ask, do we sufficiently estimate the privilege of living in an age so wonderfully progressive in scientific research and discovery? So stupendous in its works of art? Distance has been annihilated, electricity utilized by the power of the mind and the application of science through cables, which ere long will girdle the whole earth, acquainting us with events transpiring in other parts of the world, with thousands of miles of ocean intervening, in advance as it were of their actual occurrence. Rivers are spanned with bridges, which in their construction are marvels of art and science. Mountains are perforated to yield a common highway for commercial intercourse, nation with nation, all the results of science, so tremendously grand and sublime that half a century since a mere conception of the idea of a possibility of such completion would have subjected the originator to the charge of madness, if nothing worse. Contemplating all these things, one is *now* almost tempted to believe that the word impossibility will eventually be expunged from the scientific dictionary.

Having finished and brought to a successful close the prescribed course of lectures, and having passed through the ordeal of a searching examination by your faculty, with credit to yourselves and honor to your teachers, and being made "Doctors of Dental Surgery" (which means that you have not only mastered all the science appertaining to your department, but that you are competent to teach the same to others), you might naturally infer that your studies are finished, and that nothing remains for you but to carry well digested theories into practice. My dear young friends, you are perhaps ready and well prepared to *enter* the great university of practical life, in which, your diploma will be but like your matriculation ticket, obtained on entering upon your first course of lectures in this college. All your time spent in the dissecting rooms, laboratory and lecture room, together with the knowledge gained there, will be to you but practical lessons, showing you how to systematize study, and conduct investigations of details to the best advantage.

"To live, is but to learn."

Your future success as dentists, your standing among professional brethren, and your position in society, will be advantageously promoted, by a diligent course of self improvement. I know full well that to impress this upon your mind, your faculty have spared no pains. You have learned here, if not elsewhere, the meaning of education, and that it is not the work of a year, or years—but of a lifetime. Let us indulge therefore in the justifiable hope, that you will press forward, and keep pace with the times—to pause, is to go backward. There is no such thing as standing still. Let your motto be: "Onward and upward."

Forget not, that in receiving a diploma from this school, you incur high moral obligations, so to exercise and cultivate your talents, that you too, in time, may contribute your share to the common stock of knowledge. Bounteously you have received, freely and cheerfully ought you to give. See to it, that you keep bright the fame of your "alma mater," and add to the lustre of her alumni.

The advantages of associations and combined labor, you will soon learn to appreciate, especially will this be the case, if your future location is more or less remote from the great centers of communication, and when personal intercourse with your more experienced professional brethren is restricted to a few accidental meetings with one another; dental societies will then become as necessary to meet your intellectual wants, as are our depots for the supply of those of a material nature. You can not afford to be absent from such meetings. Your loss of time and outlay of money will be amply repaid by the knowledge you will gain from the varied experience of your confreres. You will be benefited by listening to, and taking part in their discussions. Flint and steel are passive in themselves, but by clashing them together fiery sparks are drawn forth; even a couple of dry sticks, nothing in themselves, being rubbed vigorously together may set your house on fire. The best intellect, isolated and left entirely to itself, must of necessity contract, become one sided in its views, if not pedantic; whereas, the wholesome attrition of different mental organizations, brightens the intellect and enlarges the understanding. The beneficial influences of association affect in a great measure, your moral and physical natures, cleansing you of narrow jealousy, strengthening your confidence in your fellow man, making you less selfish, and causing you to feel that you are co-laborers with many noble and warm hearted brethren, in the good work of elevating your profession—with men, whose mere personal acquaintance is an honor, and whose friendship is a precious boon.

Our dental literature, has not closed its recruiting offices; volunteers are always acceptable, and your duty in this respect is very plain. Accord it your earnest and cheerful support, by word and deed, by precept and example, in any and every way, give your assistance to the faithful few who are now bearing the whole burden.

Your intercourse with members of the profession, should be characterized by a spirit of unlimited liberality. There should be no secrets hid, nor any improvements or discoveries locked up. Remember, that all the knowledge you possess or may acquire, belongs to the common stock; so act, that you may have the approval of your own consciences, as worthy stewards of a great trust. Kindness, and manly frankness, will make you many friends, and any service or assistance which you may render to a brother in difficulty, will be like the sowing of good seed—you may count upon a bountiful harvest. If called upon to give your opinion upon the merits or demerits of dental operations of your fellow practitioners, your duty towards your patients and the public, demands that you impartially judge the case upon its merits, and candidly state your conclusions—remembering always, that the modifying influence of circumstances, existing at the time of, or subsequent to the operation, ought to be taken into consideration, and your final opinion should be given in a manner, least calculated to injure the reputation of your brother; neither insinuate by hints or inuendoes aught, which may be construed to his disadvantage. To sum up the whole matter, and to give you the key to the "code of ethics," do unto others, as you would have others do unto you.

Thus far, gentlemen, have I had in my mind, the worthy and honorable members of our profession, and my remarks apply to the relations which you are expected to sustain towards them alone. There are others to whom I must allude. We unfortunately, (as in other professions) have many unenlightened people among us; while there can be no congeniality of feeling, or similitude of aspirations between yourselves and the persistent and willful ignoramus, you yet must make a distinction between such and he, who does not glory in his deficiencies. To the latter you will extend the helping hand, and by kind and persevering efforts bring him "from darkness to light." The opinionated and self-conceited quack, however, is an unmitigated nuisance, and as such to be shunned. My advice to you would be to avoid the whole class—to "let them alone, *most severely*." You are always at a disadvantage with these creatures, and have no weapons with which to fight them. They are professionally outlawed, and not amenable to God or man.

Having thus summarily disposed of "Doctors" Blow-much, Know-little and Cheat-well, let us return to subjects more agreeable, inviting our attention.

You all hope in due course of time, to obtain a lucrative practice, and with it, to enjoy in the highest degree, the confidence and esteem of your patients. The most skillful, the most accomplished operator, if deficient in those attributes which make the gentleman, will fail most greviously in the realization of his hopes in this latter respect.

Your relation towards your patients brings you at once into intimate contact with them. Hence the necessity for the exercise of manly frankness, tempered with delicate courtesy. Kind attention to the comfort of those to whom you are ministering, entirely exempt from unbecoming familiarity, should characterize your daily walk. To a man or woman capable of judging character, there is nothing perhaps, so disgusting as a fawning sycophant. Guard well your speech, never let a word be spoken to a lady which the whole world might not hear. Consider your office sacred ground, and let it not be profaned.

While performing painful operations, you are oftentimes compelled to witness exhibitions of the weakness of poor human nature. The most delicate and refined, are at times so unstrung by fear and suffering, as to do injustice to their real characters. Operations, which at one time they may have borne with admirable fortitude, may at another tax them beyond their powers of endurance. Scenes of a highly ludicrous nature sometimes will occur, tempting you to an after description, but forbear—and by so doing, preserve the sanctity of your office.

I might here allude to the fact, that some patients, particularly ladies, are extremely sensitive on the subject of wearing artificial dentures; and I caution you against the too common practice of referring one patient to another. This you have no right to do, without first obtaining permission.

Your office and waiting room should be homelike and pleasant. You *can not* probably in the beginning, have your furnishings and adornments what your taste might dictate, but your rooms *can* always be orderly and cheerful, and to secure this, will require constant vigilance on your part.

Good taste and propriety should characterize your attire, together with the most scrupulous cleanliness, and in the latter respect, you *can not* be *too* fastidious in your office manipulations.

I might continue at considerable length in these "practical hints," and still leave much unsaid; general suggestions must take the place of positive rules. A Chesterfield or D'Orsay would fail to provide you with a code of etiquette for all occasions which might arise. Your instinct as true and honorable gentlemen, and that indefinable something called tact, must alone be your guide.

Your professional services will often be called into requisition for children, and in this connection every faculty of a kind heart and patient disposition, will find ample scope for exercise. Deal honestly with the child. Injudicious parents and friends will tempt you to deceive them, and take advantage of their trusting natures. Never consent to such fraud. Tell them the plain truth, explain to them the benefit to be derived from the operation—try to inspire them with courage, and wait their time. You will in almost *every case* succeed, and what is better, will have secured the confidence and trust of that little one. If your lives are spared, you may in a few years be called upon to perform similar operations for this child's children. Think then what a co-adjutor you will have in the father or mother, and what weight their own experience will have with their children.

Your able preceptors, who are also practitioners, have doubtlessly laid before you all your duties, in a far more comprehensive way, in your daily lessons, and you may deem these suggestions superfluous—but, as you are here to-night, publicly to take upon yourselves new characters, it seems meet, that you should hear once more, a short recapitulation of the most important of the responsibilities which you are about to assume.

And now, ladies and gentlemen, you who at once represent the friends of dental education and the public at large, permit me to offer a few observations for your kind consideration. You have heard me "lay down the law" to these young gentlemen, have heard me enumerate their duties towards you—did it ever occur to you, that you have imposed upon *you* duties towards *them?* If I have been at all able to make my meaning plain, you have heard what the profession requires of its members, and this college of its graduates, that you may be faithfully and intelligently served, and that you may not be left to the impositions and frauds of ignorant and unscrupulous pretenders. Your duty to these young graduates, and your own interests go hand in hand.

I would respectfully ask of you how it is, that in our very midst, the young, thoroughly educated dental surgeon, skilled and expert as an operator, and conscientious as a man, is obliged to spend the best years of his life in convincing you that he deserves your trust and confidence; whilst on the other hand, quackery is allowed to establish its headquarters side by side with our dental and medical colleges, and this on short notice? Strangers in our cities find it incomprehensible, that the hanging out of a great red or blue banner, making extravagant use of paint, together with a few shallow "tricks of the trade," (gift enterprises, etc.,) are sufficient to impose upon the credulity of the public, establish a notoriety, and command a good pecuniary harvest for those so engaged.

That these things are facts, is a disgrace to the age in which we live. It must be discouraging to young men who have undergone years of severe training in acquiring a thorough professional and scientific education, to know that as far as large portions of our communities are concerned, one who may have tried perhaps a half dozen different business pursuits in twice as many months, can as a last resort, establish a dental office in Cincinnati, (for instance) put a teakettle on the stove, or run a toy steam engine, and call it "Grand Steam Dental Establishment," and by no inconsiderable portion of your people, be considered the equal, if not the superior, of the modest and retiring man of merit.

This state of things ought not to exist, and I fear that it is a want of proper discrimination on your part, which makes you ready victims to these charlatans, thereby causing you to do an injustice, both to yourselves and to those who would serve you honestly and intelligently.

As a representative of the dental profession, I now appeal to you, and urge you to be careful in the choice of your dentist, satisfy yourselves beyond a doubt, of the man's professional attainments and antecedents, by the recommendations of friends whom you know to be capable of judging. I consider it your duty ladies and gentlemen, to give hearty and cheerful support to the meritorious, and discountenance all pretension and imposition.

Returning to you my young friends. If from what I have heretofore said, you have gathered that all is labor, care, and vexation in your future walk, without gratifying compensation, I must hasten to disabuse your minds of the idea. It is well that you enter upon your career with the shoals and quicksands upon your chart, "forewarned is forearmed." If you understand yourselves and conscietiously do your duty, on many occasions you will be cheered by the delighted manifestations of your patients, of their full appreciation of your services as rendered. Though not your province to watch with anxious solicitude, by the bedside of the suffering; to "count the ebbing strokes of life's failing tide;" to be the oracle of hope or despair. You will have daily opportunities of mitigating human suffering; and this, in itself, is a noble work. *Your* responsibilities are as nothing compared with those of the physician. You can at all times have the satisfaction of knowing whether you have benefited your patients, and have done your whole duty by them. On the other hand, the physician is oftentimes in doubt himself, often receiving commendation when none is due, and more frequently being censured when all praise had been merited.

Go forth then, with all the buoyant hopes of happy youth, and may success attend you, surpassing even your most sanguine expectations.

The appropriate gift of your faculty, accompanying each diploma, proves that as pupils, your spiritual wants have not been unheeded. In this "book of books," you will find unerring rules for your guidance—support for your faith. Profit by, and make them your own; and when life shall close, may you have that consciousness of duty performed, which will evidence to self-approving consciences, that you have not lived in vain.

In behalf of our profession, I bid you a hearty welcome to its ranks. We take pleasure in hailing you as brothers and fellows.

As the sad hour of parting draws near, it devolves upon me, on behalf of your faculty to bid you farewell, and while so doing, I would remind you, that every picture, however sombre, has its softening and relieving tints.

We part but for a season, looking forward with hope to future re-unions.

